# Electrophysiological measurements of holistic processing of Chinese characters

**DOI:** 10.3389/fpsyg.2022.976568

**Published:** 2022-08-22

**Authors:** Zhengyang Qi, Wenbo Luo

**Affiliations:** ^1^Research Center of Brain and Cognitive Neuroscience, Liaoning Normal University, Dalian, China; ^2^Key Laboratory of Brain and Cognitive Neuroscience, Liaoning Province, Dalian, China

**Keywords:** Chinese character recognition, holistic processing, composite paradigm, event-related potentials, time-frequency analysis

## Abstract

Holistic processing (HP) is a marker of perceptual expertise in facial recognition. In the present study, we examined neural responses to the HP of Chinese characters, adopting the composite paradigm. The behavioral results showed that the discrimination of congruent trials was significantly higher than that of incongruent trials, and participants responded faster. Moreover, the congruent trials elicited significantly larger N170 amplitude than the incongruent trials. The HP effect of the N170 component was observed for upright characters, as the configural information of inverted characters and misaligned characters were destroyed. Right-lateralization of processing Chinese characters was observed in the N170 amplitudes and delta-theta band oscillations. The results suggested that Chinese character recognition employed a strategy of HP, and the finding that neural indicators provide a better signal of the strength of HP in Chinese characters than behavioral indicators was also crucial.

## Introduction

Holistic processing (HP) is the tendency to process objects as a whole rather than as their parts, and is regarded as a behavioral hallmark of face recognition (Wong and Gauthier, 2010; Tso et al., 2013). The face inversion effect can be explained by HP. When faces are inverted such that the local information is constant, people find it difficult to identify inverted faces resulting from the use of holistic shape representations (Farah et al., 1995). Accumulated evidence has shown the application of HP to other types of expert-level object recognition beyond face perception, including English words (Anstis, 2005; Wong et al., 2011), Portuguese words (Ventura et al., 2019, 2020), musical notations (Wong and Gauthier, 2010), fingerprints (Busey and Vanderkolk, 2005), chessboards (Bilalić et al., 2011), and novel objects, such as Greebles and Ziggerins (Richler et al., 2009; Wong et al., 2009).

The composite paradigm is widely used to investigate the HP of face recognition (Rezlescu et al., 2017). HP is greater interfered with by the irrelevant half in a parts-matching task, meaning that performance is better when the responses to the bottom and top halves are matched (congruent trial) than when responses to the bottom and top halves are mismatched (incongruent trial) (Bukach et al., 2006). For example, when participants are quickly presented with two faces, in which the upper part is the same and the lower part is different, and they needed to determine whether the upper parts of the two faces are the same or not, their answers are always different. While, when the halves are misaligned, the discrimination to attended half of a face is less affected by the irrelevant half. Note that the complete composite design (including no misalignment condition) assesses HP by a congruency effect indicated by the performance difference between congruent and incongruent trials. In contrast, the HP in the partial design is measured by an alignment effect (i.e., the performance difference between aligned and misaligned trials) (Hsiao and Cottrell, 2009; Liu et al., 2016; Tso et al., 2020).

Given that Chinese characters share many visual properties with faces, researchers revealed HP of Chinese characters employing the composite task. Hsiao and Cottrell (2009) found that HP effect was observed among the novices, but not the experts, they suggested that HP was not a marker of general visual expertise. Dyslexic readers showed a stronger HP effect in recognizing Chinese characters than the typical readers (Tso et al., 2019, 2020, 2021; Brady et al., 2021). The reduction in HP associated with expert Chinese character recognition could be better explained by writing rather than reading experience (Tso et al., 2011, 2012, 2013, 2014). While Wong et al. (2011) and Chen et al. (2013) adopted a composite matching task, it was found that both English and Chinese characters were represented holistically at a perceptual level. Wong et al. (2012) also found that both experts and novices showed HP, irrespective of the character structure (left-right or top-bottom) or presentation sequence (sequential or simultaneous matching). Leung (2012) reported a significant positive correlation between the HP of Chinese characters and the number of years of studying Chinese. Whether HP applies to Chinese characters, especially for Chinese experts, or whether HP of Chinese characters is modulated by experimental paradigms and tasks, remains controversial. As research tools continue to advance, more attempts should be made to explain the HP patterns of Chinese characters from a cognitive-neural perspective, but this aspect is currently lacking.

In the current study, we adopted the composite paradigm to examine the HP of Chinese characters using the event-related potential (ERP) technique. Additionally, we included inverted character condition so that we also investigated the degree of destruction of configural information between the misaligned and inverted characters. It is a 2 (congruency: congruent vs. incongruent) × 3 (orientation: upright vs. misaligned vs. inverted) within-subjects experimental design. The ERP results targeted P1 and N170 components associated with early perceptual processing. P1 is a positive component peaking at approximately 100 ms after stimulus onset around the lateral occipital channels, is sensitive to the physical information of stimuli, and can be modulated by attention (Itier and Taylor, 2004; Rossion and Jacques, 2008). The N170 component, appearing as early as 130–200 ms on the surface of the occipitotemporal cortex and peaking at about 170 ms, reflects competition between faces and other expert-level objects (Rossion et al., 2007, 2004; Fan et al., 2016, 2015), and is modulated by the observer’s identification level of Chinese characters (Fan et al., 2015; Qi et al., 2016). In addition, we also measured transient modulations of the ongoing oscillatory EEG activity. Investigating time domain and time-frequency domain changes may reveal the novel neural mechanisms involved in the HP of Chinese characters.

## Materials and methods

### Participants

Fifteen native Chinese speakers (6 males, mean age = 23 years, range from 21 to 28 years old) without any neurological or psychiatric disorders, were recruited to this experiment in exchange for a payment. They were all right-handed and had normal or corrected- to- normal vision. All participants were provided informed written consent prior to the study. The study was approved by Liaoning Normal University Human Research Institutional Review Board in accordance with the Declaration of Helsinki.

### Materials

**FIGURE 1:**
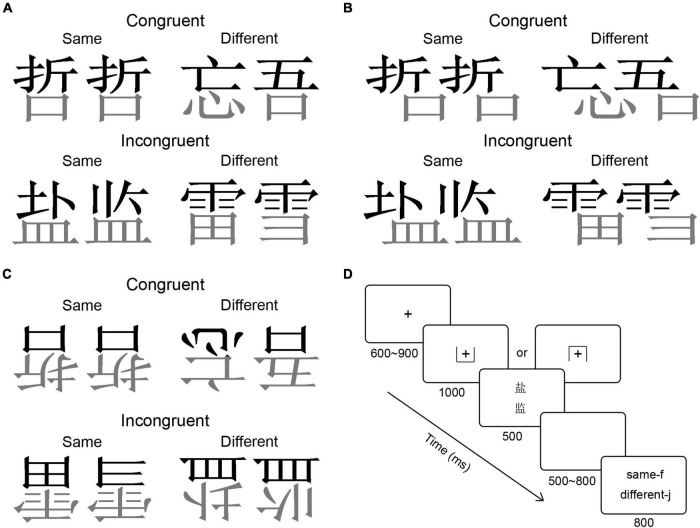
Illustration of the stimulus pairs in the composite paradigm [**(A)** for upright Chinese characters, **(B)** for misaligned Chinese characters, and **(C)** for inverted Chinese characters] and the schematic representation of the experimental procedure **(D)**.

A total of 80 pairs (four conditions of 20 pairs each) of Chinese characters with top-bottom configuration were presented in Song font. The selected characters could be horizontally divided into two components so that each pair of components to be attended to and compared appeared in one congruent and one incongruent trial. Lexical characteristics were well controlled and there were no significant differences in the number of strokes or frequency (Liu et al., 1990) between the selected characters, *ps* > 0.09. The orientation of the presentation of the Chinese character was upright (Figure 1A), misaligned (Figure 1B), or inverted (Figure 1C). All images were processed using MATLAB 2012a software to ensure that they measured 7.5 cm in width (equal to 3.77° when viewed 114 cm from the monitor) and 8.5 cm in length.

### Procedure

The experimental procedure was programmed with E-Prime 1.0 (Psychology Software Tool, Inc., Pittsburgh, PA, United States), six blocks in total, and each block had 80 trials. A trial was initiated with a fixation at the center of the screen for 600∼900 ms, followed by a 1,000 ms cue indicating participants to attention top or bottom of each character, and then 500 ms of two Chinese characters were presented above and below the initial fixation, after that, a blank screen for 500∼800 ms appeared to avoid the expecting effect. Finally, the response page was presented for 800 ms, and the intertrial interval (ITA) was 700 ms (Figure 1D). Participants were asked to judge the half of two characters that they need to attention to was the same or different as quickly and accurately as possible by pressing “f” for the same and “j” for the different. The response button has been balanced. Participants were comfortably seated in a dim and sound-isolated room, 114 cm away from the center of the screen. They performed a practice session with Chinese characters that were not used in the experiment properly.

### Behavioral data analysis

The A’, a bias-free non-parametric measure of sensitivity (Stanislaw and Todorov, 1999), was calculated as the dependent variable of discrimination performance. ${\text{A}}^{{}^{\prime }}=0.5+\left[\text{s}\,i\,g\,n\,\left(\text{H}-\text{F}\right)\,\frac{{\left(H-F\right)}^{2}+\left|H-F\right|}{4\,m\,a\,x\,\left(H,F\right)-4\,H\,F}\right]$, where H and F are the hit rate and false alarm rate, respectively. The value of A’ varies between 0.5 and 1.0, and a higher A’ indicates better discrimination. The A’ difference between incongruent and congruent trials measures HP–a more positive value marks a stronger HP effect (Tso et al., 2013).

A two-way repeated-measures analysis of variance (rmANOVA) was performed on A’ and response times (RTs) to test the effects of “Congruency” (Congruent vs. Incongruent) and “Orientation” (Upright vs. Misaligned vs. Inverted). *P*-values were corrected using the Greenhous-Geisser method, and partial eta-squared (η${}_{p}^{2}$) was reported to demonstrate the effect size in ANOVA tests.

### Electrophysiological data recording

Brain electrical activity was recorded by a 64-channel system composed of tin electrodes mounted in an elastic cap (Brain Product, Germany) according to the international 10–20 System, with FCz reference. The activity of vertical electrooculography (EOG) was collected from electrodes placed below the left eye, and the activity of horizontal EOG was recorded *via* electrodes fixed on the outer canthi of both eyes. The impedance of all electrodes was kept below 5kΩ. Electroencephalogram (EEG) and EOG signals were continuously recorded with a sampling rate of 500 Hz, filtered *via* a 0.1–100 Hz bandpass.

### Electrophysiological data analysis

#### Analysis in the time domain

Electroencephalogram data were analyzed using EEGLAB (Delorme and Makeig, 2004), off-line filtered with a 0.1∼30 Hz bandpass, and re-referenced to a common average. The EEG was segmented into epochs of 1,200 ms, including a 200 ms pre-stimulus baseline. After baseline correction, trials contaminated by gross artifacts were rejected when the amplitude of any electrode exceeded ± 100 μV. The remaining EOG artifacts were corrected using independent component analysis (ICA) (Delorme et al., 2007). Only correctly performed trials were included in the analysis.

Based on the topographical distribution of grand-averaged ERPs and previous studies (Wang et al., 2011; Luo et al., 2013), a set of electrodes were chosen to measure P1 and N170 components. P1 amplitude (98∼120 ms) was analyzed at P7/8, PO3/4, PO7/8 electrodes; N170 amplitude (160∼200 ms) was analyzed at P7/8, PO7/8 electrodes. For each component, a three-way rmANOVA was performed with the following variables as within-subject factors: “Hemisphere” (Left vs. Right), “Congruency” (Congruent vs. Incongruent), “Orientation” (Upright vs. Misaligned vs. Inverted).

#### Analysis in the time-frequency domain

The time-frequency distribution of each EEG trial was obtained using the windowed Fourier transform (WFT), in which a fixed Hanning window with a duration of 200 ms was used. For each trial, the WFT translated EEG responses into a complex time-frequency spectral estimate with the explored frequencies ranging from 1 to 30 Hz in 1 Hz steps, and with the explored latencies ranging from −200 to 1,000 ms in 2 ms steps. For each estimated frequency, results were displayed as an event-related increase or decrease in oscillation amplitude relative to a pre-stimulus reference interval (−150 to −50 ms before the stimulus onset) using the unbiased subtraction approach (Hu et al., 2014a).

Considering the topographical distribution of oscillatory activities, two time-frequency region-of-interests (ROIs) were defined in the TFDs, as follows: ROI1, delta-theta band oscillations (1∼8 Hz, 50∼300 ms, measured from P5/6, P7/8, PO7/8 electrodes); ROI2, beta-band oscillations (14∼21 Hz, 160∼400 ms, measured from P1/2, P3/4, P5/6 electrodes). For each ROI, the oscillatory magnitude was obtained by calculating the mean magnitude within the pre-defined ROI (Hu et al., 2014b). For the oscillatory magnitude within each ROI, a three-way rmANOVA was performed with the following variables as within-subject factors: “Hemisphere” (Left vs. Right), “Congruency” (Congruent vs. Incongruent), “Orientation” (Upright vs. Misaligned vs. Inverted).

## Results

### Behavioral results

For A’ (mean ± SD), the main effects of “Congruency” [*F*_(1, 14)_ = 11.86, *p* = 0.004, η${}_{p}^{2}$ = 0.46] and “Orientation” [*F*_(2, 28)_ = 6.66, *p* = 0.015, η${}_{p}^{2}$ = 0.32] were significant. Pairwise comparisons demonstrated that the discrimination capacity of participants to congruent trials (*A*’ = 0.97 ± 0.02) was significantly higher than incongruent trials (*A*’ = 0.93 ± 0.05). In addition, the discrimination capacity of participants to misaligned characters (*A*’ = 0.97 ± 0.04) was significantly higher than inverted characters (*A*’ = 0.93 ± 0.05, *p* = 0.018), while there was no difference between misaligned characters and upright characters (*A*’ = 0.96 ± 0.04, *p* = 0.35), or between upright characters and inverted characters (*p* = 0.16).

**FIGURE 2:**
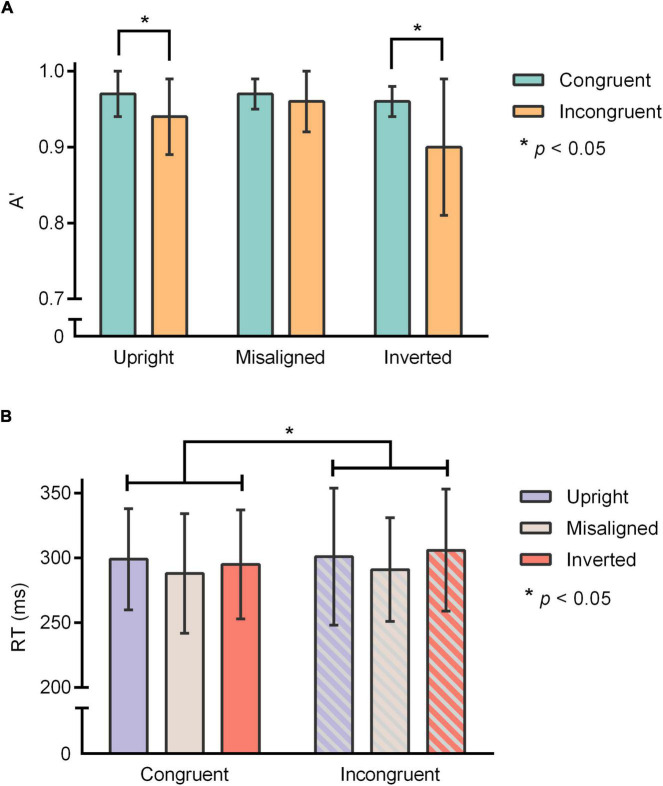
**(A)** Discrimination sensitivity (A’) in the composite task for upright, misaligned, and inverted Chinese characters. **(B)** Response time on congruent trials and incongruent trials. *p < 0.05.

Furthermore, A’ also showed a significant interaction between “Congruency” and “Orientation” [*F*_(2, 28)_ = 6.21, *p* = 0.018, η${}_{p}^{2}$ = 0.31]. Simple effect analysis demonstrated HP effect among the upright characters (*p* = 0.006) and the inverted characters (*p* = 0.008), but not the misaligned characters (*p* = 0.16) (Figure 2A).

In terms of RTs (mean ± SD), the analyses reported were carried out on correct response only. The main effects of “Congruency” [*F*_(1, 14)_ = 5.03, *p* = 0.042, η${}_{p}^{2}$ = 0.26] and “Orientation” [*F*_(2, 28)_ = 5.33, *p* = 0.011, η${}_{p}^{2}$ = 0.28] were significant, while their interaction [*F*_(2, 28)_ = 0.99, *p* = 0.37, η${}_{p}^{2}$ = 0.07] was not significant. Pairwise comparisons demonstrated that participants reacted to congruent trials (294 ± 43 ms) significantly faster than incongruent trials (299 ± 46 ms) (Figure 2B). In addition, participants reacted to misaligned characters (290 ± 43 ms) significantly faster than upright characters (298 ± 46 ms, *p* = 0.04) and inverted characters (300 ± 43 ms, *p* = 0.043), while there was no difference between upright characters and inverted characters (*p* = 1.00).

### Electrophysiological results in the time domain

**FIGURE 3:**
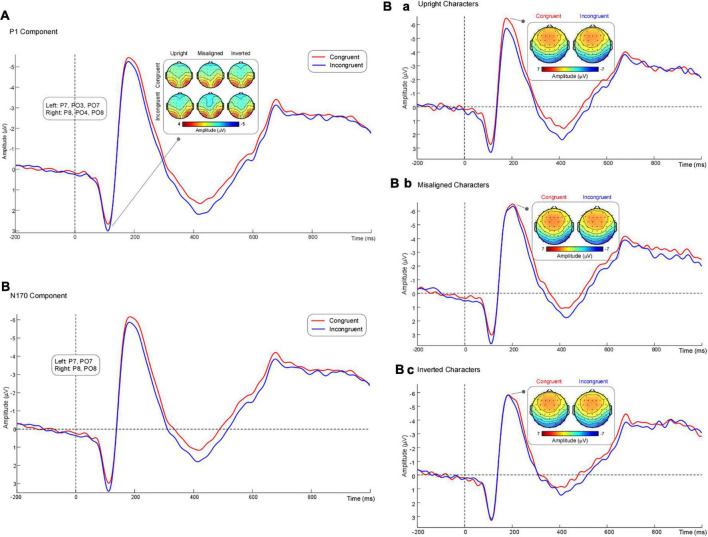
**(A)** Grand average P1 waveform and scalp topographies. **(B)** Grand average N170 waveform. Average N170 waveforms and scalp topographies elicited by congruent trials and incongruent trials for upright **(Ba)**, misaligned **(Bb)**, and inverted **(Bc)** Chinese characters, respectively.

P1 amplitude (mean ± SE) revealed significant main effects of “Congruency” [*F*_(1, 14)_ = 5.03, *p* = 0.042, η${}_{p}^{2}$ = 0.26] and “Orientation” [*F*_(2, 28)_ = 3.48, *p* = 0.045, η${}_{p}^{2}$ = 0.20]. Pairwise comparisons indicated that incongruent trials (2.80 ± 0.62 μV) elicited significantly larger P1 amplitude than congruent trials (2.52 ± 0.66 μV) (Figure 3A). In addition, according to the pairwise comparisons of orientation, the P1 amplitude elicited by upright characters (2.50 ± 0.63 μV), inverted characters (2.67 ± 0.65 μV), and misaligned characters (2.82 ± 0.64 μV) were not significant in pairs (*p* > 0.069).

N170 amplitude (mean ± SE) revealed significant main effects of “Hemisphere” [*F*_(1, 14)_ = 8.04, *p* = 0.013, η${}_{p}^{2}$ = 0.37] and “Congruency” [*F*_(1, 14)_ = 11.44, *p* = 0.004, η${}_{p}^{2}$ = 0.45]. Pairwise comparisons indicated that N170 amplitude was significantly larger in the right hemisphere (−6.61 ± 0.78 μV) than in the left hemisphere (−4.85 ± 0.55 μV). In addition, congruent trials (−5.89 ± 0.60 μV) elicited significantly larger N170 amplitude than incongruent trials (−5.58 ± 0.61 μV) (Figure 3B).

There was a significant interaction between “Congruency” and “Orientation” [*F*_(2, 28)_ = 4.18, *p* = 0.031, η${}_{p}^{2}$ = 0.23]. Simple effect analysis indicated that congruent trials (−6.20 ± 0.52 μV) elicited significantly larger N170 amplitude than incongruent trials (−5.42 ± 0.56 μV, *p* < 0.001) among the upright characters (Figure 3Ba), while there was no significant difference among the misaligned characters (*p* = 0.56) (Figure 3Bb) or the inverted characters (*p* = 0.76) (Figure 3Bc).

### Electrophysiological results in the time-frequency domain

The magnitude of delta-theta band oscillations (ROI1) displayed significant main effects of “Hemisphere” [*F*_(1, 14)_ = 13.64, *p* = 0.002, η${}_{p}^{2}$ = 0.49] and “Orientation” [*F*_(2, 28)_ = 7.78, *p* = 0.006, η${}_{p}^{2}$ = 0.36]. Pairwise comparisons indicated that ROI1 magnitude was significantly larger in the right hemisphere (0.72 ± 0.12 μV) than in the left hemisphere (0.38 ± 0.06 μV). In addition, both upright characters (0.55 ± 0.08 μV, *p* = 0.044) and misaligned characters (0.62 ± 0.10 μV, *p* = 0.013) evoked significantly larger magnitude than inverted characters (0.49 ± 0.08 μV), while there was no significant difference between them (*p* = 0.22).

**FIGURE 4:**
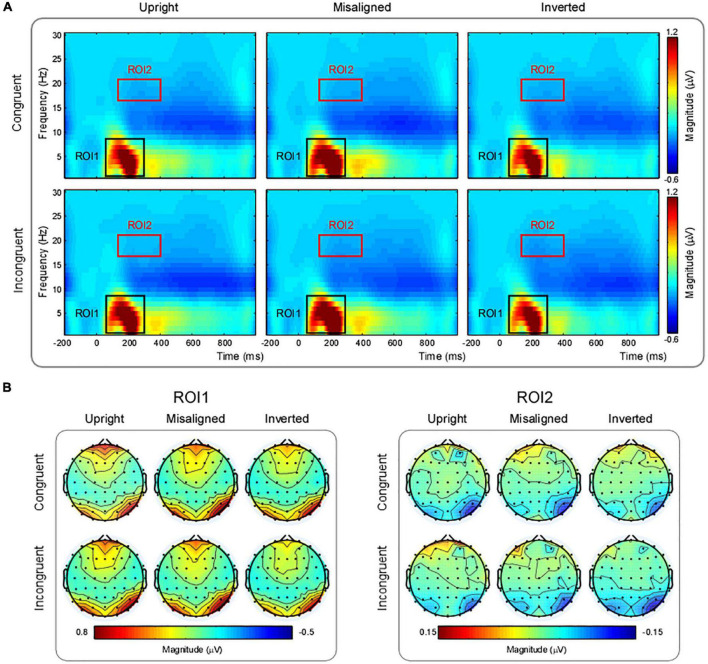
**(A)** Grand-average time-frequency representations in different experimental conditions. **(B)** Scalp topographies of time-frequency oscillations in two ROIs for each experimental condition.

ROI2: beta-band oscillations. There was a significant interaction between “Hemisphere” and “Congruency” [*F*_(1, 14)_ = 9.04, *p* = 0.009, η${}_{p}^{2}$ = 0.39]. Simple effect analysis indicated that incongruent trials (−0.04 ± 0.01 μV) evoked significantly larger magnitude than congruent trials (−0.03 ± 0.01 μV, *p* = 0.005) in the left hemisphere, while there was no significant difference in the right hemisphere (*p* = 0.63) (Figure 4).

## Discussion

In this study, using the composite paradigm, we observed the neural mechanisms underlying the HP of Chinese characters. Our results can be summarized as follows: First, HP is the way in which Chinese characters that are observed at the behavioral level are recognized. The target parts of two characters are interfered with by the irrelevant parts, and such interference is reduced when the parts are misaligned and inverted. At the electrophysiological level, incongruent trials elicited larger P1 amplitudes than congruent trials, while congruent trials elicited larger N170 amplitudes than incongruent trials. This HP effect was only observed among upright characters and absent among misaligned and inverted characters because of the destruction of configural information. Second, we did not observe the inversion of Chinese characters, which may have been influenced by the experimental paradigm and task. Lastly, there was a right-hemisphere lateralized advantage in Chinese character recognition in that larger N170 amplitudes and delta-theta band magnitudes were observed in the right hemisphere than in the left hemisphere.

### Behavioral and electrophysiological evidence for holistic processing of Chinese characters

Holistic processing has been associated with perceptual expertise in alphabetic (Coltheart et al., 1993; Anstis, 2005; Wong et al., 2011) and non-alphabetic languages (Tso et al., 2011; Chen et al., 2013). Our behavioral results are comparable with that HP is a marker of expertise for Chinese characters (Wong et al., 2012). This study identified an interaction between orientation and congruency, with significant HP effects for upright and inverted characters, but no significant HP effects for misaligned characters. In terms of RTs, subjects recognized misaligned characters significantly faster than upright and inverted ones. This suggests that the degree of destruction of configural information is greatest for misaligned characters, as no HP effect was found for either discrimination or reaction time. Although inverted characters also destroy the configural information between the components of the characters, their configural information is preserved to a greater extent than that of misaligned characters. Thus, subjects are hindered in adopting HP, but this hindrance is less than in the case of misaligned characters.

At the electrophysiological level, we also found that Chinese characters were processed holistically during the different time courses. P1 component was generally considered to be influenced by attention distribution and was greatly influenced by physical properties of stimuli. The results of the present study showed that incongruent trials elicited a larger waveform of the P1 component than congruent trials, which is thought to be significantly influenced by stimulus contrast and whose amplitude is modulated by attention, whereas incongruent trials elicited attention first at the early visual processing stage. The incongruent trials were more difficult to identify than the congruent trials and caught people’s attention in the early stages of visual processing.

However, congruent trials elicited greater N170 amplitudes than incongruent trials, suggesting that the N170 responds to the holistic nature of Chinese character processing. This was particularly confirmed by the interaction between orientation and congruency, where the configural information in misaligned and inverted characters is disrupted. Thus, no HP effect is produced, and only in upright characters is a HP effect significant, thus also revealing that brain neural activity is more sensitive to the configural information of Chinese characters. HP is a marker of expertise; therefore, congruent trials elicited a larger N170 than incongruent ones, indicating that N170 reflects the characteristics of the HP of Chinese characters. The HP effect was only observed among upright characters, and not among misaligned and inverted ones, indicating that the neural activity of the brain is more sensitive to the HP of Chinese characters.

Chen et al. (2013) adopting the adaptation paradigm reported that P1 was smaller when the attended top parts were the same compared with when they were different, which was similar to our P1 results. While they reported this effect was present only when the two character parts were aligned but not misaligned, and only for characters but not for pseudo-characters. Our results indicated a comparable effect reflected in the N170 component. The adapting stimulus in their adaptation paradigm was displayed for about 2,800 ms, and the stimulus in the current study was presented for 800 ms. Rapid response to stimulus occupied more attention resources, resulting in HP reflected as elaborate processing.

Regarding the time-frequency results, we found that incongruent trials evoked larger beta-band magnitudes than congruent trials in the left hemisphere. Beta-band oscillations are considered to be aware of motor processes in action words, attention, inhibition, memory, and binding mechanisms during language processing (Weiss and Mueller, 2012). The functional characteristics of beta-band oscillations are mainly thought to be related to motor activity, which shows a significant decrease in the beta band when the main motor cortical movement is present but produces a strong beta energy rebound when the movement stops. In the current study, beta-band oscillations were measured starting at 160 ms, which was behind the P1 component (ended at 120 ms). Therefore, in incongruent trials with enhanced beta energy evoked at later times, there is a possibility of attentional return or the continuation of P1 results.

### Right hemisphere lateralization of Chinese characters

Holistic processing has been generally thought to be associated with the right hemisphere. Ventura et al. (2018) found that the right hemisphere played a bigger role in holistic word processing than the left hemisphere. While recent behavioral evidence showed that HP and right hemisphere lateralization do not necessarily go together, depending on the task requirements (Hsiao and Cheung, 2011; Chung et al., 2018; Tso et al., 2020, 2017). There was no direct relationship between HP and the right hemisphere, we just observed a right-hemisphere advantage of Chinese character processing in that the N170 amplitude and delta-theta band oscillations were larger in the right hemisphere than in the left hemisphere. This result is consistent with the study by Wang et al. (2011), who found that the amplitude elicited by composite characters showed right hemisphere lateralization, but the latencies were bilateral. And Mousavi et al. (2020) reported that the delta and theta band both increased in the right parietal lobe during verbal fluency tasks. The lateralization of the brain induced by Chinese characters is unclear. Researchers have reported that both real and pseudo-characters elicited left-hemisphere lateralization of the N170 response (Cao et al., 2011), and emotional words induced left-hemisphere lateralization (Zhang et al., 2014; Yi et al., 2015). And some studies found bilateral hemispheric elicited by Chinese characters, without hemispheric differences (Zhang et al., 2011). There is still controversy regarding the lateralization induced by Chinese characters, which needs to be further explored.

### Inversion effect of Chinese characters

In this study, we added inverted characters to our investigation of the HP of Chinese characters, and we were also able to investigate the inversion effect of Chinese characters. According to the results, a distinct inversion effect was not observed. There was a main effect of “Orientation,” which showed that the discrimination of misaligned characters was higher than inverted characters, and participants responded faster to misaligned characters than they did to upright and inverted characters. As mentioned previously, this phenomenon may be the configural information of misaligned characters damaged maximally; therefore, it would be easy for participants to distinguish them. However, we found that upright and misaligned characters evoked larger delta-theta band oscillations than inverted characters. Delta oscillations are a collection of a wide range of cortices that are responsible for selective attention and language processing based on syntax (Schürmann et al., 2001; Roehm et al., 2004). Theta oscillations are responsible for myriad cognitive activities such as declarative memory processing, successful memory encoding, consolidation of the memory of a large amount of information, episodic memory, and processing (Sauseng and Klimesch, 2008). Delta-theta band oscillations contain a lot of information related to memory, and it can be speculated that upright and misaligned characters that contain semantic information are easier to save in memory. However, it is not easy to involve semantic information and memory processing in the early stages of inverted character processing, which results in a reduction in delta-theta band oscillations. Furthermore, the stimuli were presented by a pair of Chinese characters in our study, while other studies showed the inversion effect of Chinese characters by presenting single Chinese characters in orientation judgment tasks (Wang et al., 2011). It is unclear whether a pair of Chinese characters can produce an inversion effect that may be related to experimental stimuli and tasks.

## Data availability statement

The raw data supporting the conclusions of this article will be made available by the authors, without undue reservation.

## Ethics statement

The studies involving human participants were reviewed and approved by the Liaoning Normal University Human Research Institutional Review Board. The patients/participants provided their written informed consent to participate in this study.

## Author contributions

ZQ and WL contributed to conception and design of the study. ZQ organized the database and performed the statistical analysis, wrote the first draft of the manuscript, and wrote sections of the manuscript. WL contributed to manuscript revision, read, and approved the submitted version. Both authors contributed to the article and approved the submitted version.
